# Patient‐driven search for rare disease therapies: the Fondazione Telethon success story and the strategy leading to Strimvelis

**DOI:** 10.15252/emmm.201607293

**Published:** 2017-02-01

**Authors:** Lucia Monaco, Lucia Faccio

**Affiliations:** ^1^ Fondazione TelethonMilanItaly

**Keywords:** Genetics, Gene Therapy & Genetic Disease

## Abstract

The recent approval of Strimvelis, the first *ex vivo* gene therapy to gain marketing authorization (Schimmer & Breazzano, 2016), has drawn attention to Fondazione Telethon, the Italian charity that played a pivotal role in this effort. Although it is not uncommon that advanced therapies, such as Strimvelis, are developed by partnerships between academia and industry, direct involvement of a charity in key steps of this process is still unusual. Illustrating the strategies and operational model adopted by Fondazione Telethon to achieve its mission of supporting excellent research aimed at curing rare genetic diseases may elucidate some of the enabling factors behind the Strimvelis success story.

## About Telethon's research

Fondazione Telethon was founded in 1990 at the behest of a group of patients, who expressed the need for an independent organization in Italy to support research on rare genetic diseases, such as muscular dystrophies. As a charity, Fondazione Telethon financially relies mainly on donations from various fundraising initiatives, which started with a television marathon that still takes place in December each year on Italian public TV.

The central activity of Fondazione Telethon to fulfill its mission is research on rare genetic diseases. Two fundamental principles are applied throughout any funding decisions: all research must be scientifically excellent, and it must respond to the patients' mandate to find and make available therapies for their diseases. Scientific excellence is maintained through selection procedures that comply with internationally recognized standards of peer review; the selection process is managed by dedicated professionals with years of experience in biomedical research and a strong scientific background to safeguard competence, fairness and transparency. Fondazione Telethon's peer review model is one of the key factors behind the success of its research; it is considered a benchmark in Italy, and was cited as a model of good practice (Jurkat‐Rott & Lehmann‐Horn, 2004; Pammolli *et al*, 2009).

## A growing research portfolio

Soon after it was founded, Fondazione Telethon realized that a composite set of funding initiatives, each responding to different research needs and opportunities, would best serve its goals. As the initial annual research budget of 9.5 billion Italian lira (4.9 million Euros) progressively increased to the current amount of around 30 million Euros, the foundation was able to expand its funding from the initial call for extramural research projects in 1991 to intramural research, including the Telethon Institute of Genetics and Medicine (TIGEM) founded in 1994, the San Raffaele‐Telethon Institute for Gene Therapy (SR‐TIGET) founded in 1995 in partnership with the San Raffaele hospital in Milan, and the Dulbecco Telethon Institute, the virtual institute assembling the awardees of the Telethon career program, launched in 1999. In all cases, a scientific advisory board of outstanding scientists and patient associations' representatives constantly assesses the research portfolio and provides strategic advice on funding.

**Figure 1:**
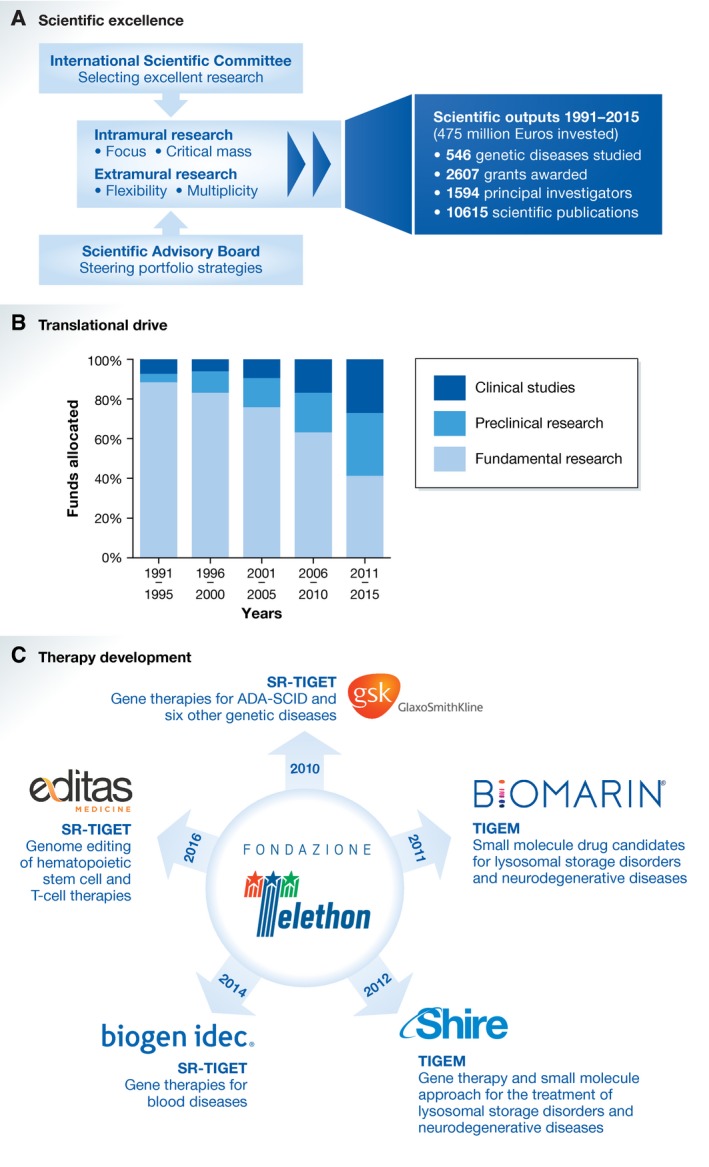
The pillars of Fondazione Telethon's commitment to research (A) Scientific excellence is safeguarded by the rigorous and transparent selection of the best research projects by an international scientific committee and by the steering of the research portfolio by an expert scientific advisory board, which contribute to the constant increase of Fondazione Telethon's scientific outputs. (B) Translational drive is exerted by constantly steering research toward clinical applications and has gradually shifted research investments from fundamental research to pre‐clinical and clinical studies. Research costs exclude unallocable costs such as intramural fixed costs, open‐access fees. Source: Fondazione Telethon's TRic database. (C) The development of therapies requires partnerships with biotech/pharma companies. Five major alliances have been achieved by Fondazione Telethon in the past 6 years for the major research areas in its intramural institutes SR‐TIGET and TIGEM. For SR‐TIGET, partnerships are co‐signed by Fondazione Telethon and the San Raffaele Hospital in Milan.

The two lines of intramural and extramural research offer different and often complementary opportunities to tackle rare genetic diseases, and imply different roles for the foundation. Extramural research, which is funded through competitive calls, allows supporting the best ideas in a flexible fashion, through 1‐ to 3‐year grants awarded to principal investigators in Italian non‐profit research institutes. Much of Fondazione Telethon's basic research, addressing hundreds of genetic diseases, has thus been supported. In contrast, intramural research allows focusing on specific diseases and activities, achieving and maintaining critical mass, and guarantees continuity for strategic lines of research (Ballabio & Naldini, 2015) (Fig 1A).

## Monitoring and developing research outputs

As any scientific funding body, Fondazione Telethon constantly monitors and keeps track of its research investments and scientific outputs by its investigators (Fig 1A), and relays them back to all stakeholders by issuing yearly balance sheets, and through regular scientific conventions and meetings with patient advocacy groups.

Journal impact factor or other bibliometric indexes are not considered in the *ex ante* evaluation of research proposals, but Fondazione Telethon runs aggregate *ex post* analyses based on article citations (Thomson Reuters, 2011; Naik, 2016), which demonstrate that the overall quality of its scientific research is comparable to that of top international research institutes.

In addition to scientific publications, other outcomes are required from Telethon‐funded projects. Investigators are encouraged to pursue development of their research to the clinical level, which implies fostering collaborations with the clinic, safeguarding intellectual property, and actively pursuing partnerships with pharmaceutical and biotech companies.

## The path to therapies

The first step to foster translational research is increasing researcher motivation to directly benefit the rare disease patients. Any Telethon project, even basic research, must therefore be clearly linked to a genetic disease and bear a potential impact on patients' lives. This tenet is specifically addressed in the peer review process: for instance, all projects that undergo competitive evaluation receive a specific “impact on patients” score, which is combined with the scientific merit score in the final ranking. Projects with a higher impact on patients gain an advantage over projects with equal scientific merit but farther away from a direct benefit for patients.

It is through the intramural institutes, however, that Fondazione Telethon plays a more direct role in developing research results up to patient fruition through intellectual property protection and technology transfer activities that enable strategic partnerships with biotech companies and pharmaceutical industries. Here again, a professional team manages intellectual property, scouting for and negotiating industrial partnerships, managing industrial alliances, and conducting regulatory activities. Owing to these strategies, Fondazione Telethon's investment has progressively shifted from basic to translational research (Fig 1B), up to the point where therapeutic approaches that have been successful in pre‐clinical or clinical testing require the involvement of biotech/pharma companies to make these available for patients.

Unlike common diseases, rare diseases cannot rely on extensive experience of developing drugs and therapies beyond the clinical proof of concept, or on large investments by the pharmaceutical sector: indeed, the so‐called “valley of death” between clinical results and approved therapies is particularly wide and deep. Fondazione Telethon therefore does not consider its duties completed until all possible steps are made to bridge this gap, starting from IP protection and acquiring orphan drug designation to facilitating interactions with regulatory authorities, to ensure that studies will be conducted in compliance with good clinical practices and meet all regulatory requirements for marketing approval. This *modus operandi* makes Fondazione Telethon‐funded research appealing for its industrial partners, which is vital to perform safe and standardized production of therapies after completion of clinical studies. The success of this holistic strategy may be exemplified by the development of Strimvelis.

## Gene therapy: the ADA‐SCID case

By founding an institute dedicated to gene therapy in 1995, Fondazione Telethon seized the opportunities of this new field. SR‐TIGET, created in association with Ospedale San Raffaele, a major Italian scientific and clinical research hospital, allowed the recruitment of excellent scientists committed to developing gene delivery platforms, in collaboration with clinicians dedicated to the study of disease natural history and to fine‐tuning the delivery options for the new therapies. Fondazione Telethon also took a primary role in the key steps of clinical development, such as investing in the development and production of clinical‐grade viral vectors for *ex vivo* gene therapy; acquiring orphan drug designations from EMA and the US FDA; requesting protocol assistance from EMA to define in advance how to design the clinical study to achieve solid data for registration; and supporting the creation of the first academic laboratory certified with good laboratory practices for cell and gene therapy. This strong commitment proved crucial for maintaining the focus in the critical years after the cases of leukemia in gene therapy clinical trials on X‐SCID (Hacein‐Bey‐Abina *et al*, 2003). Indeed, while research and clinical studies aimed at treating ADA‐SCID with gene therapy continued, the institute promptly started a new line of research to determine the genotoxicity of the treatment, which is a prerequisite for any gene therapy trial today.

By the end of 2009, the ADA‐SCID clinical trial had generated convincing proof of safety and efficacy in 12 patients (Aiuti *et al*, 2009). However, to give all ADA‐SCID patients access required resources, facilities, and competences that are typical of biotech/pharma companies: a daunting need, given the extreme rarity of the disease. Fondazione Telethon soon realized that partnering with a large company would be necessary, not only to bring the ADA‐SCID therapy to the market but also to develop a whole technology platform encompassing the other diseases in the SR‐TIGET clinical pipeline, which would enable the company to tackle more diseases.

The partnership signed by Fondazione Telethon and the San Raffaele hospital with GlaxoSmithKline (GSK) in 2010 created an alliance that led to the approval of Strimvelis and the development of a research pipeline to find cures for six more diseases. As of today, GSK has also an exclusive license for gene therapies to cure metachromatic leukodystrophy and the Wiskott–Aldrich syndrome and is moving toward submission for marketing authorization.

## Moving forward

Since its agreement with GSK, Fondazione Telethon has entered four more major industrial partnerships to develop the results of front‐line research on genetic diseases in the TIGEM and SR‐TIGET institutes (Fig 1C). In all cases, Fondazione Telethon's investigators preserve their independence and Fondazione Telethon retains the intellectual property that is licensed to the partner. To safeguard the benefit to patients, the industrial partners are contractually bound to develop the therapies up to patient availability or return any intellectual property and results co‐developed to Fondazione Telethon. Importantly, these partnerships also include funding in support of research, and Fondazione Telethon invests any profits from royalties into research.

Industrial partnerships are again being monitored through dedicated alliance managers whom Fondazione Telethon has recruited to manage the multifaceted interaction between investigators and clinicians in the institutes and the companies' research teams. The Telethon Clinical Development Unit has also been created to facilitate the development of all clinical operations in collaboration with the clinical team in the San Raffaele Hospital in Milan and with two major hospitals in Naples that are involved in the TIGEM clinical programs. The unit coordinates activities by, and facilitates interactions among the study teams; organizes clinical meetings; and manages any contracts with clinical research organizations, clinical manufacturing organizations, and external laboratories required for pre‐clinical, manufacturing, or clinical operations.

Finally, Fondazione Telethon's direct engagement in regulatory activities, particularly those related to advanced therapies, has set an example of the pivotal role of non‐profit organizations among all the official actors needed for product development, which should be acknowledged by the regulatory authorities. Notwithstanding its growing commitment to developing effective therapies for people affected by genetic diseases, Fondazione Telethon is not relenting on its investment on fundamental research, which is the basis on which any future therapies will be developed. This poses a challenge of striking the right balance in distributing research investments across the research portfolio to accomplish maximum benefit for patients with the available resources.

## Conflict of interest

The authors declare that they have no conflict of interest.
